#  Características contextuais e procura por serviços de saúde entre adolescentes brasileiros: Pesquisa Nacional de Saúde, 2019

**DOI:** 10.1590/0102-311XPT070223

**Published:** 2023-12-11

**Authors:** Larissa Adna Neves Silva, Bruno Pereira Nunes, Juliana Gagno Lima, Elaine Tomasi, Luiz Augusto Facchini

**Affiliations:** 1 Programa de Pós-graduação em Epidemiologia, Universidade Federal de Pelotas, Pelotas, Brasil.; 2 Faculdade de Enfermagem, Universidade Federal de Pelotas, Pelotas, Brasil.; 3 Instituto de Saúde Coletiva, Universidade Federal do Oeste do Pará, Santarém, Brasil.

**Keywords:** Serviços de Saúde, Adolescentes, Iniquidades em Saúde, Estudos Transversais, Health Services, Adolescents, Health Inequalities, Cross-Sectional Studies, Servicios de Salud, Adolescentes, Inequidades en Salud, Estudios Transversales

## Abstract

Os objetivos foram descrever a prevalência de procura por serviços de saúde entre adolescentes brasileiros e investigar sua associação com características contextuais do território. O estudo utilizou dados da *Pesquisa Nacional de Saúde* de 2019, realizada com 43.774 indivíduos de 10 a 19 anos. A informação do adolescente foi obtida por meio de um morador *proxy* de 18 anos ou mais que respondia por si e por todos os moradores da casa. A regressão de Poisson foi utilizada para avaliar a procura por serviços de saúde de acordo com região geopolítica, situação censitária e tipo de município. Também foi avaliada a interação da variável “plano de saúde” nessas associações. Do total, 11,7% (IC95%: 11,1; 12,3) dos adolescentes procuraram serviços de saúde nas duas semanas anteriores à pesquisa. Maiores prevalências de procura foram observadas nas regiões Sudeste (RP = 1,32; IC95%: 1,15; 1,52) e Sul (RP = 1,31; IC95%: 1,13; 1,52) em comparação à Região Norte do país. O acesso a plano de saúde aumentou a busca pelos serviços por adolescentes residentes nas áreas rurais e nas capitais e municípios das Regiões Metropolitanas e/ou Regiões Integradas de Desenvolvimento. O estudo evidenciou baixa prevalência de procura por serviços de saúde entre adolescentes e desigualdades contextuais para a região geopolítica. Ter plano de saúde foi um marcador importante para entender as disparidades na situação censitária e no tipo de município.

## Abstract (en)

This study aimed to describe the prevalence of demand for health services among Brazilian adolescents and to investigate its association with contextual characteristics of the territory. Study with data from the *Brazilian National Health Survey*, conducted in 2019, including 43,774 individuals aged from 10 to 19 years. Adolescent’s information was obtained through a proxy resident of 18 years or more who answered for all the residents of the household. Poisson regression was used to assess the demand for health services according to geopolitical region, economic status, and type of municipality. The interaction of the health insurance was also evaluated in these associations. Of the total, 11.7% (95%CI: 11.1; 12.3) of the adolescents sought health services in the two weeks prior to the survey. Greater figures of demand were observed in the Southeast (PR = 1.32; 95%CI: 1.15; 1.52) and South regions (PR = 1.31; 95%CI: 1.13; 1.52) compared to the Northern Region of Brazil. Having a health insurance increased the demand for services by adolescents living in rural areas and in capitals and the municipalities of the Metropolitan Areas and/or Integrated Development Regions. The study showed a low prevalence of demand for health services among adolescents and contextual inequalities for the geopolitical region. Having a health insurance was an important marker to understand the disparities in the economic status and in the type of municipality.

## Resumen

El objetivo de este estudio fue estimar la prevalencia de la demanda de servicios de salud entre los adolescentes brasileños e investigar su asociación con las características contextuales del territorio. Estudio con datos de la *Encuesta Nacional de Salud*, realizada en 2019 con 43.774 individuos de entre 10 y 19 años de edad. La información del adolescente se obtuvo de un residente *proxy* de 18 años o más que era responsable de sí mismo y de todos los residentes de la casa. La regresión de Poisson se utilizó para evaluar la demanda de servicios de salud según la región geopolítica, la situación en el censo y el tipo de municipio. También se evaluó la interacción de la variable seguro médico en estas asociaciones. Del total, el 11,7% (IC95%: 11,1; 12,3) de los adolescentes buscaron servicios de salud en las dos semanas previas a la encuesta. Se observó mayor prevalencia de demanda en las regiones Sudeste (RP = 1,32; IC95%: 1,15; 1,52) y Sur (RP = 1,31; IC95%: 1,13; 1,52) en comparación con el Norte del país. Tener un seguro médico incrementó la búsqueda de servicios por parte de los adolescentes que viven en áreas rurales y en las capitales y municipios de de las Regiones Metropolitanas y/o Regiones de Desarrollo Integrado. El estudio apuntó a una baja prevalencia de demanda de servicios de salud entre los adolescentes y desigualdades contextuales según la región geopolítica. Tener un seguro médico fue un marcador importante para comprender las disparidades según la situación del censo y el tipo de municipio.

## Introdução

A adolescência é o período de mudanças fisiológicas e psicológicas significativas na transição da infância para a idade adulta, considerado um momento importante para estabelecer uma boa saúde ^1^. Apesar de avaliada como uma fase saudável da vida, estima-se que 1,1 milhão de adolescentes entre 10 e 19 anos morrem a cada ano. As principais causas são acidentes de trânsito, suicídio e violência interpessoal ^2^.

A maior parte da mortalidade e morbidade que ocorre na adolescência é evitável ou tratável, mas os adolescentes enfrentam barreiras específicas no acesso a informações e serviços de saúde ^2^. Esses obstáculos estão relacionados, principalmente, a questões financeiras e geográficas, como longas distâncias até o serviço, inexistência de ações específicas para atender esse público, falhas no acolhimento, falta de confidencialidade e atitudes negativas dos profissionais de saúde diante do adolescente, motivadas pelo desconhecimento, desinteresse ou ainda por uma visão estigmatizada sobre a adolescência ^3^^,^^4^^,^^5^^,^^6^. Isso leva à menor probabilidade de adolescentes terem acesso aos cuidados de saúde em comparação às pessoas de outras faixas etárias ^7^^,^^8^.

Estudo nacional identificou que 56,7% de adolescentes escolares procuraram os serviços de saúde nos últimos 12 meses ^9^. A menor procura dos serviços de saúde tem sido associada aos adolescentes socialmente desfavorecidos ou marginalizados, com piores níveis socioeconômicos e que moram em regiões menos desenvolvidas ^9^^,^^10^^,^^11^^,^^12^^,^^13^. Ademais, algumas pesquisas mostram que o atendimento geralmente acontece em consultórios particulares ou em unidades básicas de saúde (UBS), com motivos relacionados à busca por vacinação, a consultas de rotina ou devido a algum problema de saúde ^9^^,^^14^.

De modo geral, o acesso aos serviços de saúde depende da procura, que, por sua vez, é influenciada pela situação de saúde do indivíduo, pela percepção de necessidades e por suas características sociodemográficas ^10^. O acesso também depende da oferta e da disponibilidade dos serviços de saúde, que estão relacionadas a marcantes desigualdades regionais no Brasil ^15^. Uma revisão sistemática demonstrou a existência de diversos estudos brasileiros sobre acesso e utilização de serviços de saúde ^7^, no entanto, as análises sobre adolescentes são pouco frequentes ^9^^,^^11^^,^^12^^,^^13^^,^^14^^,^^15^. Ainda, a maioria é voltada para populações específicas, como escolares ^9^^,^^11^^,^^12^^,^^13^, adolescentes atendidos por determinado tipo de serviço (como saúde bucal ou saúde mental) ^3^, nível de atenção ^14^ ou adolescentes que residiam apenas na área urbana ^15^.

Também são escassas as evidências nacionais sobre acesso aos serviços por adolescentes. Somente dois estudos com dados da *Pesquisa Nacional de Saúde do Escolar* (PeNSE) foram encontrados ^9^^,^^11^. Além disso, os resultados desses trabalhos demonstraram disparidades regionais, evidenciando a necessidade de mais pesquisas que considerem também os adolescentes que não estão nas escolas e que esclareçam essas diferenças entre as regiões e outros aspectos contextuais.

Diante da necessidade do reconhecimento das desigualdades para desenvolver ações estratégicas e políticas públicas de saúde direcionadas a esse público, este estudo teve como objetivos descrever a prevalência da procura por serviços de saúde entre adolescentes brasileiros e investigar sua associação com características contextuais do território.

## Métodos

Trata-se de um estudo transversal, de base populacional, com dados da *Pesquisa Nacional de Saúde* (PNS) realizada entre agosto de 2019 e março de 2020 pelo Instituto Brasileiro de Geografia e Estatística (IBGE) em parceria com o Ministério da Saúde. A amostra da PNS foi representativa da população brasileira residente em domicílios particulares situados em áreas urbanas e rurais, nas cinco grandes regiões geopolíticas, 27 Unidades da Federação (UF), capitais e Regiões Metropolitanas. O processo amostral foi realizado em três estágios: os setores censitários foram as unidades primárias de amostragem, seguidos dos domicílios e, por último, dos indivíduos ^16^.

A coleta de dados foi feita por entrevistadores treinados por meio de dispositivos móveis de coleta. A PNS utilizou três questionários: um referente às informações do domicílio; outro sobre todos os moradores da residência; e o terceiro direcionado ao morador com 15 anos de idade ou mais selecionado para participar da pesquisa. Para este trabalho, foram utilizadas informações dos dois primeiros questionários, em que os respondentes eram indivíduos com 18 anos de idade ou mais, que respondiam por si e pelos demais moradores do domicílio. Assim, as informações dos sujeitos incluídos neste estudo, adolescentes com 10 a 19 anos de idade, foram relatadas por esse indivíduo com 18 anos de idade ou mais que residia na mesma casa que os adolescentes durante o período da coleta de dados. Mais detalhes sobre processo de amostragem e instrumentos utilizados pela PNS podem ser encontrados em estudo metodológico ^16^.

Considerou-se como desfecho a procura por serviços de saúde entre adolescentes, obtido por meio da pergunta: “Nas duas últimas semanas, você procurou algum lugar, serviço ou profissional de saúde para atendimento relacionado à própria saúde?” (N = 43.774). As exposições principais foram as características contextuais: região geopolítica (Centro-oeste; Nordeste; Norte; Sudeste; Sul); tipo de situação censitária (urbana; rural); e tipo de município (capital; Região Metropolitana e Região Integrada de Desenvolvimento - RIDE; demais municípios).

A delimitação das Regiões Metropolitanas e RIDEs ocorre por meio de legislações específicas, como determinado na *Constituição Federal* de 1988 (art. 21 e 25). Enquanto as Regiões Metropolitanas são formadas por agrupamentos de municípios limítrofes do próprio estado e são instituídas por lei complementar estadual, as RIDEs são definidas como regiões administrativas que abrangem municípios de diferentes estados e, portanto, a competência de criá-las é da União. O objetivo principal do IBGE ao disponibilizar dados a partir desses recortes territoriais está na visualização das particularidades dessas regiões em razão das necessidades de planejamento e gestão, assim como na identificação dos municípios que compõem esses agrupamentos ^17^. Atualmente, no Brasil, existem três RIDEs e 74 Regiões Metropolitanas ^18^.

Para fins de ajuste, incluímos as seguintes variáveis independentes:

(i) Cobertura de atenção à saúde: domicílio cadastrado na unidade de saúde da família (USF) (sim; não) e acesso a plano de saúde (sim; não);

(ii) Características sociodemográficas: sexo (masculino; feminino); idade (anos completos); cor ou raça (branca; preta; parda; amarela/indígena); frequenta a escola (não; sim, na rede pública; sim, na rede privada); e renda familiar *per capita* em salários mínimos (até 1/4; mais de 1/4 até 1/2; mais de 1/2 até 1; mais de 1 até 2; mais de 2 até 3; mais de 3 até 5; e mais de 5);

(iii) Características da situação de saúde: estado de saúde do adolescente, segundo a percepção do respondente sobre a saúde do adolescente (muito bom; bom; regular; ruim/muito ruim); nas duas últimas semanas, deixou de realizar quaisquer de suas atividades habituais - trabalhar, ir à escola, brincar, afazeres domésticos etc. - por motivo da própria saúde (sim; não); diagnóstico médico de alguma doença crônica, física ou mental, ou doença de longa duração, de mais de seis meses (sim; não).

A base de dados foi obtida na página de Internet do IBGE (http://www.ibge.gov.br). As análises estatísticas foram realizadas no pacote Stata, versão 15.0 (https://www.stata.com), considerando o desenho amostral do estudo por meio do módulo *survey*. Os gráficos foram criados no Microsoft Excel 2019 (https://products.office.com/). Inicialmente, realizou-se uma análise descritiva da cobertura de saúde e das características contextuais, sociodemográficas e de situação de saúde por meio de frequências absolutas e proporções. Também se estimou a prevalência da procura por serviços de saúde entre os adolescentes brasileiros e de acordo com as variáveis independentes, assim como seus respectivos intervalos de 95% de confiança (IC95%), com as diferenças avaliadas pelo teste qui-quadrado de heterogeneidade.

Para explorar a associação entre a procura dos serviços de saúde e as características contextuais, foram calculados modelos brutos e ajustados por meio da regressão de Poisson, obtendo-se as razões de prevalência (RP) e respectivos IC95%, além de valores de p por meio do teste de Wald de heterogeneidade. Para isso, construiu-se um modelo hierárquico utilizando o procedimento de *backward selection* por níveis e mantendo as variáveis que apresentaram valor de p < 0,2 no nível do modelo ajustado.

Inicialmente, foram estimados separadamente o efeito da região, o tipo de situação censitária e o tipo de município sobre o desfecho. No nível 1, essas características contextuais foram inseridas ao mesmo tempo no modelo. No nível 2, a variável “domicílio cadastrado na USF” foi adicionada ao modelo, mas ela não permaneceu por apresentar valor de p > 0,2.

No nível 3, foram adicionadas as variáveis que representaram as características sociodemográficas (sexo; idade; cor ou raça; frequenta a escola; e renda familiar *per capita*). No nível 4, foi incluída a variável “plano de saúde”; e no nível 5, foram inseridas as características de situação de saúde (percepção do respondente sobre a saúde do adolescente; nas duas últimas semanas deixou de realizar quaisquer de suas atividades habituais; e diagnóstico médico de alguma doença crônica, física ou mental, ou doença de longa duração).

Em um último ajuste, testamos a interação entre plano de saúde e características contextuais. O termo de interação para região não foi significativo - por isso, foi excluído do modelo final. O teste de interação foi realizado somente para essa variável individual sob a hipótese de que o sistema de saúde brasileiro é caracterizado pela coexistência de serviços públicos e privados e considerando que, dependendo do local de residência, a oferta desses serviços pode ser diferente entre aqueles que têm possibilidade de ter plano de saúde e aqueles que não têm ^19^. Além disso, ao testar os modelos, quando essa variável foi adicionada em diferentes níveis do modelo de análise, observaram-se maiores alterações estatísticas na magnitude do efeito das características contextuais em relação ao desfecho.

Para interpretação das interações, foram estimadas as probabilidades marginais do desfecho ajustadas para as covariáveis do modelo 6 segundo as variáveis “tipo de situação censitária” e “tipo de município”, utilizando o comando margins. Para todos os testes realizados, foram consideradas estatisticamente significativas associações com valores de p < 0,05.

A PNS foi aprovada pela Comissão Nacional de Ética em Pesquisa com Seres Humanos do Ministério da Saúde (parecer nº 3.529.376). Todos os participantes assinaram termo de consentimento livre e esclarecido, que garantiu a confidencialidade dos dados.

## Resultados

Entre os adolescentes com idade de 10 a 19 anos, 38,1% pertenciam à Região Sudeste e 1/3 à Região Nordeste; 22% residiam em capitais; 18,4% em municípios das Regiões Metropolitanas e das RIDEs; e 59,6% moravam em outros municípios. A maior parte dos adolescentes era de áreas urbanas (83%); 2/3 dos adolescentes tinham o domicílio cadastrado na USF; e 1/5 tinha plano de saúde.

Com relação às características individuais, pouco mais da metade da população estudada era do sexo masculino e de cor ou raça parda; 68,4% frequentavam a escola na rede pública; e 1/3 da amostra apresentou renda familiar *per capita* de mais de 1/2 até 1 salário mínimo. Cerca de 2/3 dos entrevistados relataram que os adolescentes que residiam no seu domicílio tinham bom estado de saúde; 9,5% receberam o diagnóstico médico de alguma doença; e 5,3% deixaram de realizar atividades habituais por motivo de saúde (Tabela 1).

**Tabela 1:** Características da população estudada e prevalência de procura por serviços de saúde entre adolescentes de 10 a 19 anos, segundo as variáveis independentes. *Pesquisa Nacional de Saúde*, Brasil, 2019 (N = 43.774).

Variáveis	Descrição da amostra (N = 43.774)		Procura dos serviços de saúde (n = 4.492)		
	n	% *	%	IC95%	Valor de p **
Região					< 0,001
Norte	11.477	11,0	9,0	8,2; 10,0	
Nordeste	16.358	30,0	10,2	9,5; 10,9	
Sudeste	7.471	38,1	13,6	12,3; 15,0	
Sul	4.069	13,1	12,8	11,4; 14,3	
Centro-oeste	4.399	7,7	9,7	8,5; 11,4	
Tipo de situação censitária					< 0,001
Urbano	31.668	83,0	12,1	11,5; 12,9	
Rural	12.106	17,0	9,4	8,5; 10,3	
Tipo de município					0,0080
Capital	14.288	22,0	13,0	12,0; 14,1	
Municípios das Regiões Metropolitanas (excluindo a capital) e das RIDEs	6.939	18,4	12,6	11,1; 14,3	
Demais municípios (excluindo as Regiões Metropolitanas e as RIDEs)	22.547	59,6	10,9	10,1; 11,7	
Domicílio cadastrado na USF					0,3132
Sim	29.904	65,9	11,3	10,7; 12,1	
Não	9.064	23,1	12,5	11,2; 13,9	
Não sabe	4.806	11,0	11,9	10,1; 14,0	
Acesso a plano de saúde					< 0,001
Não	37.094	79,9	10,5	9,9; 11,1	
Sim	6.680	20,1	16,3	14,8; 18,0	
Sexo					< 0,001
Masculino	22.225	51,2	9,8	9,1; 10,6	
Feminino	21.549	48,8	13,6	12,8; 14,5	
Cor ou raça ***					0,0015
Branca	13.689	38,8	13,1	12,1; 14,1	
Preta	3.816	9,3	12,4	10,5; 14,5	
Amarela/Indígena	502	1,1	9,9	5,3; 17,7	
Parda	25.762	50,7	10,5	5,9; 11,2	
Frequenta a escola					< 0,001
Não	6.819	15,8	12,4	11,0; 13,8	
Sim, na rede privada	5.983	15,8	14,8	13,1; 16,6	
Sim, na rede pública	30.972	68,4	10,8	10,1; 11,5	
Renda familiar *per capita* (salários mínimos) ^#^					< 0,001
Até 1/4	9.606	16,7	10,4	9,2; 11,6	
Mais de 1/4 até 1/2	11.496	23,7	10,0	9,0; 11,2	
Mais de 1/2 até 1	12.422	30,6	11,8	10,9; 12,8	
Mais de 1 até 2	6.740	19,0	11,7	10,5; 13,0	
Mais de 2 até 3	1.748	4,9	15,0	12,2; 18,3	
Mais de 3 até 5	1.035	3,0	16,7	12,9; 21,4	
Mais de 5	705	2,1	23,6	18,5; 29,6	
Percepção de saúde do respondente sobre a saúde do adolescente					< 0,001
Muito bom	8.817	23,9	9,8	8,7; 11,0	
Bom	28.966	63,7	10,4	9,7; 11,1	
Regular	5.485	11,2	20,1	18,3; 22,0	
Ruim/Muito ruim	506	1,1	40,5	32,7; 48,8	
Nas duas últimas semanas, deixou de realizar quaisquer de suas atividades habituais por motivo da própria saúde					< 0,001
Não	41.596	94,7	9,1	8,6; 9,7	
Sim	2.178	5,3	57,4	53,5; 61,3	
Diagnóstico médico de alguma doença crônica, física ou mental, ou doença de longa duração					< 0,001
Não	40.212	90,5	10,3	9,8; 10,9	
Sim	3.562	9,5	24,7	22,5; 27,1	
Prevalência geral			11,7	11,1; 12,3	

IC95%: intervalo de 95% de confiança; RIDE: Região Integrada de Desenvolvimento; USF: unidades de saúde da família.

* Percentual considerando o delineamento do estudo: *svy*;

** Teste qui-quadrado de heterogeneidade;

*** 5 dados faltantes;

^#^ 22 dados faltantes.

A prevalência geral de procura por serviços de saúde entre adolescentes nas duas últimas semanas anteriores à pesquisa foi de 11,7% (IC95%: 11,1; 12,3). As maiores prevalências de procura foram encontradas nas regiões Sudeste (13,6%; IC95%: 12,3; 15,0) e Sul (12,8%; IC95%: 11,4; 14,3), em áreas urbanas (12,1%; IC95%: 11,5; 12,9) e por adolescentes que residiam nas capitais (13,0%; IC95%: 12,0; 14,1) e municípios das Regiões Metropolitanas ou das RIDEs (12,6%; IC95%: 11,1; 14,3) (Tabela 1).

Também procuraram significativamente mais os serviços de saúde: adolescentes que tinham plano de saúde (16,3%; IC95%: 14,8; 18,0), eram do sexo feminino (13,6%; IC95%: 12,8; 14,5), da cor ou raça branca (13,1%; IC95%: 12,1; 14,1), estudavam em escola privada (14,8%; IC95%: 13,1; 16,6) e tinham renda familiar *per capita* de mais de 5 salários mínimos (23,6%; IC95%: 18,5%; 29,6). Além disso, a procura também foi maior para aqueles que tinham o estado de saúde ruim/muito ruim (40,5%; IC95%: 32,7; 48,8), que deixaram de realizar alguma atividade por motivos da própria saúde (57,4%; IC95%: 53,5; 61,3) e entre aqueles que referiram diagnóstico médico de alguma doença (24,7%; IC95%: 22,5; 27,1).

**Figura 1:**
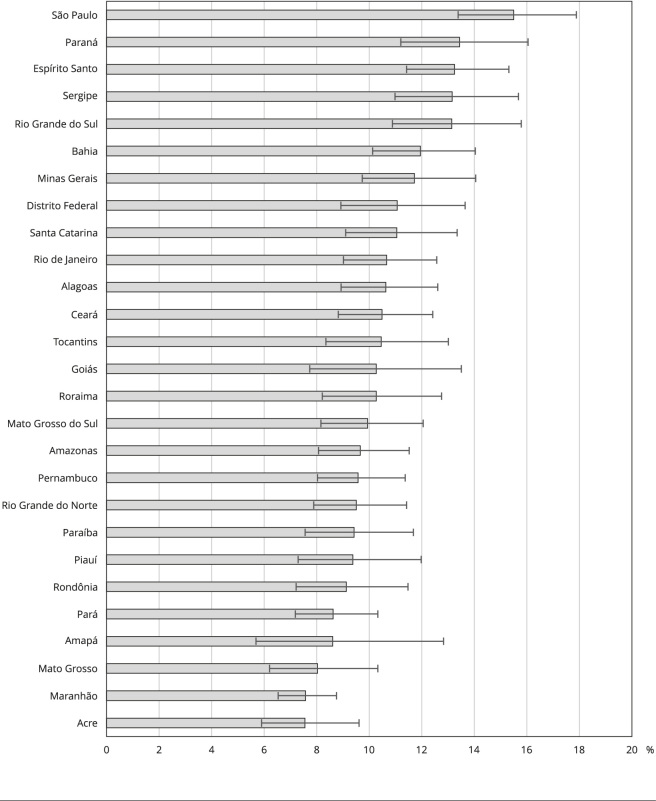
Prevalência de procura por serviços de saúde entre adolescentes, de acordo com a Unidade de Federação. *Pesquisa Nacional de Saúde*, Brasil, 2019.

A Figura 1 mostra a prevalência de procura por serviços de saúde de acordo com a UF. As UFs com maiores prevalências de procura se concentraram nas regiões Sudeste (São Paulo e Espírito Santo), Sul (Paraná e Rio Grande do Sul) e Nordeste (Sergipe e Bahia). No entanto, algumas UFs da Região Nordeste, como Maranhão e Piauí, apresentaram as menores prevalências de procura, similarmente à maioria das UFs da Região Norte. Há também um destaque para Mato Grosso, que esteve entre as UFs com menores prevalências.

A Tabela 2 apresenta os modelos brutos e ajustados da procura por serviços de saúde entre adolescentes de acordo com as características contextuais. No modelo 1, observa-se que adolescentes que residiam nas regiões Sudeste e Sul (RP = 1,50; IC95%: 1,31; 1,73 e RP = 1,41; IC95%: 1,22; 1,64, respectivamente), em áreas urbanas (RP = 1,29; IC95%: 1,16; 1,45) e nas capitais (RP = 1,19; IC95%: 1,07; 1,33) apresentaram maior probabilidade de procurar os serviços de saúde do que suas categorias de referência, mesmo quando as variáveis foram ajustadas umas para outras (modelo 2).

**Tabela 2:** Modelos brutos e ajustados da associação entre a procura por serviços de saúde entre adolescentes brasileiros e as características contextuais. *Pesquisa Nacional de Saúde*, Brasil, 2019 (n = 43.752).

Variáveis	Modelo 1 *		Modelo 2 **		Modelo 3 ***		Modelo 4 ^#^		Modelo 5 ^##^		Modelo 6 ^###^	
	RP	IC95%	RP	IC95%	RP	IC95%	RP	IC95%	RP	IC95%	RP	IC95%
Região	p < 0,001		p < 0,001		p < 0,001		p < 0,001		p < 0,001		p < 0,001	
Norte	1,00		1,00		1,00		1,00		1,00		1,00	
Nordeste	1,13	1,00; 1,27	1,15	1,01; 1,30	1,14	1,01; 1,29	1,13	1,00; 1,28	1,06	0,94; 1,19	1,05	0,94; 1,18
Sudeste	1,50	1,31; 1,73	1,46	1,27; 1,68	1,40	1,20; 1,62	1,36	1,17;1,58	1,32	1,14;1,51	1,32	1,15; 1,52
Sul	1,41	1,22; 1,64	1,42	1,22; 1,65	1,34	1,15; 1,57	1,33	1,13; 1,55	1,31	1,13;1,52	1,31	1,13; 1,52
Centro-oeste	1,09	0,91; 1,30	1,06	0,88; 1,25	1,00	0,84; 1,20	1,00	0,84; 1,20	0,99	0,94;1,16	0,98	0,83; 1,16
Tipo de situação censitária	p < 0,001		p = 0,0242		p = 0,0959		p = 0,1615		p = 0,3686		p = 0,9885	
Rural	1,00		1,00		1,00		1,00		1,00		1,00	
Urbano	1,29	1,16; 1,45	1,15	1,02; 1,30	1,12	0,98; 1,27	1,10	0,96; 1,25	1,06	0,94; 1,19	1,11	0,98; 1,26
Tipo de município	p = 0,0038		p = 0,0457		p = 0,2975		p = 0,4618		p = 0,2004		p = 0,0898	
Demais municípios (excluindo as Regiões Metropolitanas e as RIDEs)	1,00		1,00		1,00		1,00		1,00		1,00	
Municípios da Regiões Metropolitanas e das RIDEs (excluindo a capital)	1,15	1,00; 1,33	1,06	0,91; 1,24	1,05	0,91; 1,23	1,04	0,90; 1,21	1,04	0,91; 1,19	0,98	0,84; 1,15
Capital	1,19	1,07; 1,33	1,16	1,03; 1,31	1,10	0,97; 1,23	1,08	0,99; 1,23	1,10	0,99; 1,23	1,00	0,87; 1,13
Interação entre tipo de situação censitária e plano de saúde											p = 0,0334	
Sim#Urbano											0,61	0,41; 0,89
Interação entre tipo de município e plano de saúde											p = 0,0108	
Sim#Municípios das Regiões Metropolitanas e das RIDEs											1,22	0,93; 1,60
Sim#Capital											1,38	1,08; 1,76

IC95%: intervalo de 95% de confiança; RIDE: Região Integrada de Desenvolvimento.

* Modelo 1: bruto;

** Modelo 2: ajustado para as variáveis região, tipo de área e tipo de município;

*** Modelo 3: ajustado para as variáveis do modelo 2 + sexo e renda familiar;

^#^ Modelo 4: ajustado para as variáveis do modelo 3 + plano de saúde;

^##^ Modelo 5: ajustado para as variáveis do modelo 3 + estado de saúde, se deixou de realizar alguma atividade habitual por motivos da própria saúde e se algum médico deu diagnóstico de alguma doença;

^###^ Modelo 6: ajustado para as variáveis do modelo 5 + termos de interação.

Após a inclusão das características sociodemográficas (modelo 3) no modelo, as variáveis “tipo de situação censitária” e “tipo de município” deixaram de ser estatisticamente significativas. No modelo 4, após a inserção da variável “plano de saúde”, observou-se uma redução importante na magnitude do efeito da região sobre a procura por serviços de saúde em comparação ao modelo 1. Já no modelo 5, com a incorporação das variáveis de situação de saúde, identificou-se que a região permaneceu associada ao desfecho, evidenciando maiores prevalências de procura por serviços entre os adolescentes residentes nas regiões Sudeste e Sul, independentemente das outras variáveis incluídas no modelo.

No modelo 6 consideraram-se as interações entre “ter plano de saúde” e as variáveis “tipo de situação censitária” e “tipo de município”. As interações foram significativas (p < 0,05), sugerindo que a associação positiva entre tipo de situação censitária e tipo de município e a procura por serviços de saúde é modificada pela presença de plano de saúde.

**Figura 2:**
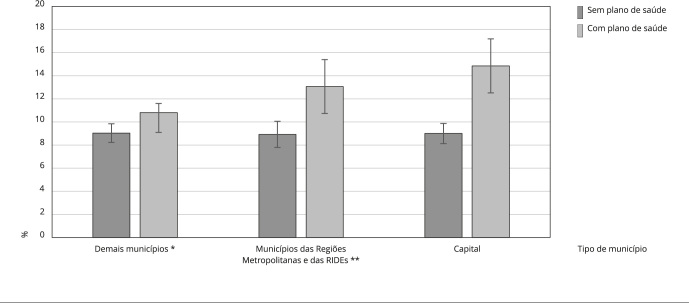
Probabilidades marginais ajustadas da procura por serviços de saúde entre adolescentes de acordo com tipo de município e acesso a plano de saúde. *Pesquisa Nacional de Saúde*, Brasil, 2019.

A Figura 2 mostra as probabilidades marginais ajustadas de procura por serviços de acordo com o tipo de município e o acesso a plano de saúde. Observou-se que a probabilidade de procura por serviços de saúde foi maior para os adolescentes que tinham algum plano de saúde, com diferenças significativamente maiores para os adolescentes que residiam em municípios das Regiões Metropolitanas e das RIDEs e em capitais. Além disso, para aqueles que não tinham plano de saúde, notou-se que a probabilidade de procura por serviços foi similar entre os tipos de município.

RIDE: Região Integrada de Desenvolvimento. * Excluindo as Regiões Metropolitanas e as RIDEs; ** Excluindo a capital.

**Figura 3:**
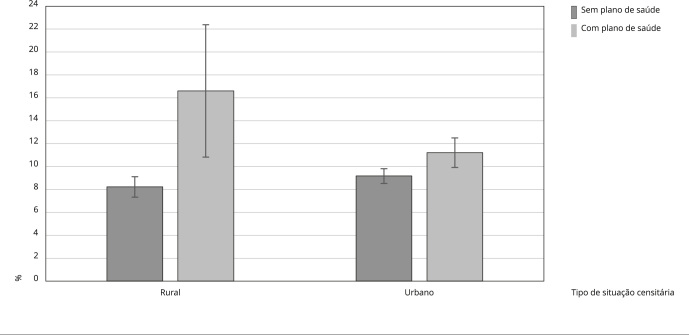
Probabilidades marginais ajustadas da procura por serviços de saúde entre adolescentes de acordo com tipo de situação censitária e acesso a plano de saúde. *Pesquisa Nacional de Saúde*, Brasil, 2019.

Na Figura 3 constam as probabilidades marginais ajustadas de procura por serviços de saúde segundo o tipo de situação censitária e o acesso a plano de saúde. Tanto na área urbana como na rural, observou-se maior probabilidade de procura por serviços entre os adolescentes que tinham algum plano de saúde em relação aos que não tinham. No entanto, a probabilidade de procura dos serviços foi duas vezes maior entre os adolescentes que moravam na área rural e tinham algum plano de saúde em relação àqueles que moravam na mesma área e não tinham plano e 47% maior em comparação aos adolescentes que moravam na área urbana e tinham algum plano de saúde.

## Discussão

Os resultados deste estudo evidenciaram uma baixa prevalência da procura por serviços de saúde: apenas um a cada dez adolescentes brasileiros (11,7%) procuraram pelo atendimento nos 15 dias anteriores à entrevista. Observou-se que os adolescentes que residiam nas regiões Sudeste e Sul procuraram em torno de 30% mais os serviços de saúde do que os adolescentes da Região Norte. Também se constatou que a presença de plano de saúde aumentou a probabilidade de procura por serviços de saúde para os adolescentes que moravam em áreas rurais e em capitais e municípios das Regiões Metropolitanas e/ou das RIDEs.

Outros estudos brasileiros encontraram prevalências diferentes de procura por serviços de saúde por adolescentes. Por exemplo, análises realizadas com dados da PeNSE demonstraram que nos anos de 2012 e 2015 as prevalências de procura nos últimos 12 meses por adolescentes escolares foram de 48% e 57%, respectivamente ^9^^,^^11^. Dados similares aos da PeNSE foram encontrados em outro estudo de base escolar (escolas da rede estadual) realizado em Olinda (Pernambuco), em 2018, considerando o mesmo período recordatório (43%) ^10^. A diferença no período recordatório contribuiu para menor prevalência observada nesse estudo em relação ao que está disponível na literatura. Autores descrevem que maiores prevalências são observadas em períodos recordatórios mais longos. Contudo, quanto maior o período recordatório, menos precisas se tornam as estimativas relatadas ^7^^,^^20^.

Em geral, o relato também depende de características individuais, que incluem capacidade cognitiva, idade, educação, situação socioeconômica, estado de saúde, entre outras variáveis que podem afetar potencialmente o processo de recordação e recolhimento de informações ^7^^,^^21^. As estimativas ainda são afetadas pelo perfil da amostra, a exemplo de adolescentes escolares ou que são atendidos por algum tipo de serviço de saúde.

Outra questão que poderia justificar as prevalências díspares neste e em outros estudos está na forma de coleta dos dados. Como a informação foi coletada por meio de um morador *proxy*, é possível que a prevalência esteja subestimada pela falta de informações importantes sobre os adolescentes, inclusive sobre a procura dos serviços de saúde, principalmente quando pode existir certa preocupação dos adolescentes em relação à privacidade e confidencialidade ^22^.

A prevalência de procura por serviços na população em estudo foi inferior àquelas encontradas em pesquisas com amostras de outros grupos etários ^7^^,^^8^. Inclusive, uma das pesquisas também realizada com dados da PNS ^8^ identificou maior procura dos serviços entre crianças e idosos em comparação à faixa etária intermediária (adolescentes e adultos jovens). Este resultado revela que ainda existem dificuldades no acompanhamento do crescimento e desenvolvimento dos adolescentes nos serviços de saúde. Além dos fatores relacionados a características organizacionais, barreiras que incluem preocupações sobre confidencialidade, desconhecimento dos serviços e desconforto em compartilhar preocupações com os profissionais levam os adolescentes a manter certo distanciamento dos serviços de saúde ^13^. Em contrapartida, quando os adolescentes procuram, as equipes de saúde apresentam dificuldades no acolhimento ^23^^,^^24^, tornando necessários mais investimentos na orientação e capacitação dos profissionais de saúde em relação a esse público e suas especificidades ^14^.

Neste estudo, observou-se maior procura por serviços nas regiões Sudeste e Sul, em contraste significativo com as regiões Centro-oeste, Nordeste e Norte, que apresentaram um padrão mais baixo, mas similar, mesmo após ajuste para várias covariáveis. É provável que esse achado exponha as desigualdades na distribuição de serviços entre as regiões, reflexo do processo de desenvolvimento histórico, político e econômico seletivo, fragmentado e desigual do Brasil ^15^.

As desigualdades no desenvolvimento socioeconômico das regiões brasileiras influenciam a distribuição de bens e serviços, em especial do setor da saúde, altamente concentrados no Sudeste e no Sul ^25^. Níveis mais elevados de infraestrutura e disponibilidade de serviços de saúde, incluindo recursos humanos, tecnológicos e mecanismos de financiamento disponíveis e adequados, são características da oferta que impactam positivamente o acesso aos serviços de saúde ^26^.

Um dos destaques da política de saúde para áreas economicamente desfavorecidas em todo o país, sobretudo nas regiões Norte e Nordeste, foi a expansão da Estratégia Saúde da Família (ESF). Neste estudo, as diferenças na procura não foram significativas em razão do cadastramento do domicílio do adolescente a uma USF. O achado sugere que a expansão da ESF promoveu resultados mais equitativos na busca por serviços, pois o esperado seria um pior desempenho, diante de seu predomínio em municípios menores e mais pobres, assim como em áreas mais vulneráveis de grandes cidades, que enfrentam maiores dificuldades de estrutura e processo de trabalho das equipes ^13^^,^^26^^,^^27^. Contudo, o cadastramento do domicílio a uma ESF não significa que o adolescente tenha vínculo com o serviço de saúde, ainda mais quando existem dificuldades de acolhimento desse público na unidade de saúde ^23^^,^^24^.

Com maior concentração de serviços de saúde nas regiões Sudeste e Sul, destaca-se que é exatamente nesses locais que a presença do setor privado é mais frequente ^25^. Em 2019, cerca de 29% e 26% dos adolescentes das regiões Sudeste e Sul, respectivamente, tinham plano de saúde, em comparação a 9,2% da Região Norte. Do mesmo modo, a prevalência de acesso a plano de saúde foi de 31,4% e 24,3% para os adolescentes que residiam em capitais e municípios das Regiões Metropolitanas ou das RIDEs, comparados aos que viviam em outros municípios (14,7%).

Ter plano de saúde pode ser um fator individual que auxilia na compreensão do acesso desigual aos serviços ^11^. Em geral, essa variável está associada positivamente a melhores níveis socioeconômicos ^19^. Observou-se que as prevalências de procura por serviços de saúde foram maiores para aqueles adolescentes em que a renda familiar *per capita* superava mais de 2 salários mínimos. Tal resultado pode estar associado à possibilidade que os indivíduos com maior renda têm de pagar por um plano de saúde, aumentando o acesso aos serviços de saúde e resultando em sobreutilização dos serviços por parte dos usuários desses planos em relação àqueles que utilizam apenas o Sistema Único de Saúde (SUS) ^28^.

Nas capitais e municípios das Regiões Metropolitanas e RIDEs, a maior procura por serviços de saúde entre adolescentes com plano de saúde pode estar relacionada à maior disponibilidade e capacidade instalada de serviços de saúde privados nessas áreas ^19^. No entanto, entre os adolescentes sem cobertura por plano de saúde, esses mesmos municípios apresentaram prevalência de procura por serviços de saúde semelhante aos adolescentes que viviam em outros municípios. Isso pode ser um indicativo da falta de serviços públicos de saúde nessas regiões, especialmente nas Regiões Metropolitanas e RIDEs. Esse é um fator preocupante, considerando que esses territórios, apesar da complexidade econômica, abrangem periferias com altas concentrações de pobreza e condições precárias e desiguais de vida ^29^, sendo prioritária a expansão da ESF e do acesso à rede pública de saúde.

As associações encontradas a partir da interação para as áreas rurais e a procura por serviços de saúde devem ser interpretadas com cautela. Na amostra geral, a prevalência de acesso a plano de saúde foi de 20,1%, sendo 3,7% nas áreas rurais e 23,5% nas áreas urbanas, corroborando estudos que evidenciam baixos percentuais de pessoas nas áreas rurais com algum tipo de plano de saúde ^19^^,^^30^.

Na área rural, é muito escassa a disponibilidade de serviços de saúde públicos e de planos de saúde. Os adolescentes do meio rural sem planos de saúde, em boa parte, são integrantes de classes trabalhadoras e populares que enfrentam barreiras de acesso e acessibilidade muito marcantes para buscar serviços públicos em outras localidades. Já os adolescentes rurais com plano de saúde geralmente pertencem a uma elite proprietária pouco numerosa e com muito poder material, que facilita a procura de serviços públicos e privados em outros lugares, às vezes muito distantes da área de residência ^31^^,^^32^^,^^33^.

Desse modo, o acesso a plano de saúde constitui um marcador de riqueza importante para entender as desigualdades em saúde no meio rural que podem estar relacionadas a determinantes estruturais, como posse da terra, bens e acesso à educação, além das características regionais ^30^^,^^33^.

Este estudo permitiu explorar a variabilidade da procura por serviços de saúde por adolescentes brasileiros, em relação às características do contexto social e dos indivíduos. Os contrastes observados entre as regiões geopolíticas do país, o tipo de situação censitária do domicílio, o tipo de município de residência e a capacidade econômica de dispor de plano de saúde são subsídios valiosos para a orientação de políticas capazes de aumentar o acesso equitativo e qualificar o vínculo dos adolescentes com os serviços públicos de saúde.
